# Efficient and Wide Chemical-Space Ionization of Organic Contaminants Using LC–MS with a Miniaturized Plasma Source Applying Different Discharge Gases

**DOI:** 10.1021/acs.analchem.5c03745

**Published:** 2025-09-16

**Authors:** Irene Caño-Carrillo, Bienvenida Gilbert-López, David Moreno-González, Joachim Franzke, Juan F. García-Reyes

**Affiliations:** † Analytical Chemistry Research Group, Department of Physical and Analytical Chemistry, 16747 Universidad de Jaén, 23071 Jaén, Spain; ‡ 28371 Leibniz Institut für Analytische Wissenschaften (ISAS e.V), 44123 Dortmund, Germany

## Abstract

Dielectric barrier discharge ionization has gained significant interest due to its versatility and broad chemical coverage. Although electrospray ionization (ESI) is the most commonly used ionization source for organic contaminant analysis by liquid chromatography–mass spectrometry (LC–MS), it has limitations such as low ionization efficiency for nonpolar compounds and matrix effects. This study investigates the potential of flexible microtube plasma (FμTP) as an alternative ionization source for the LC–MS determination of multiclass pesticides comprising ESI-amenable and organochlorine contaminants. The analytical performance of FμTP was assessed in terms of limits of quantification, reproducibility, linearity, and matrix effects, comparing the results to those obtained with ESI and atmospheric pressure chemical ionization (APCI) sources. Sensitivity assessment based on calibration slopes showed that 70% of the pesticides had higher sensitivity with FμTP than with ESI. Regarding the matrix effects, between 76 and 86% of the pesticides showed negligible matrix effects for FμTP, compared to 35–67% for ESI and 55–75% for APCI across the different matrices evaluated. The study further explored the use of argon and argon–propane mixtures as alternatives to helium as discharge gases. Results showed similar LOQs for nearly 90% of the pesticides in the positive mode and 80% of the organochlorines in the negative mode. Notably, some ion species differed when using argon-based gases for certain organochlorine pesticides, suggesting the discharge gas influences the ionization mechanism, especially in the negative mode. Overall, FμTP proves to be a sensitive and robust miniaturized ionization source, expanding the chemical space and making it useful for both target and nontarget screening applications.

## Graphical abstract

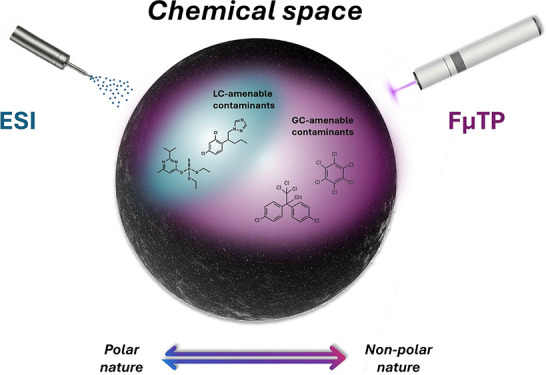

Liquid chromatography coupled with mass spectrometry (LC–MS) using electrospray ionization (ESI) is currently one of the most widely employed techniques for organic contaminants analysis due to its high sensitivity and selectivity.^1,2^ However, quantitative LC–MS analysis still presents certain challenges, one of the most significant being the matrix effects. This phenomenon arises from competition between the analyte and coeluting interfering species, leading to either signal suppression or enhancement.^3,4^ Consequently, different strategies have been developed to minimize matrix effects in LC–ESI-MS.^5^ Another well-known phenomenon that contributes to signal suppression in LC–ESI-MS is adduct formation, which complicates spectral interpretation and leads to unpredictable ionization processes with low repeatability.^6^ Multiple studies have proposed strategies to mitigate this issue, including the use of additives in the LC mobile phase.^7,8^ Furthermore, alternative ionization sources such as atmospheric pressure chemical ionization (APCI) have been investigated, as their different ionization mechanisms could help to overcome the challenges associated with sodium adduct formation in electrospray ionization, typically linked to liquid-phase surface effects.^9^

Despite these challenges, LC–MS remains the technique of choice in most small-molecule applications due to its ability to handle complex analyte mixtures,^10^ particularly for those exhibiting a polar character. It is also increasingly used for the simultaneous analysis of pesticide transformation products, which are generally more polar and less volatile than the parent compounds.^11^ However, while ESI can ionize a wide range of pesticides, other techniques such as gas chromatography coupled with mass spectrometry (GC–MS), remain essential for certain pesticide classes, including organochlorine pesticides.^12^ Both techniques are complementary, and the combined use of LC–MS and GC–MS would be required to cover the full spectrum of pesticide chemical classes. In this context, different approaches have been proposed to further enhance the capabilities of both techniques and to overcome their individual limitations. One example was the coupling of electron ionization (EI), commonly used in GC, with LC–MS systems.^13,14^ This LC–EI-MS interface showed notable potential to reduce matrix effects caused by coeluted interfering substances, while allowing the simultaneous detection of compounds that are difficult to ionize by ESI-MS, such as organochlorine and organophosphorus pesticides.^15,16^

Nevertheless, it is important to note that no single method can detect or identify chemicals across the complete scope of the so-called chemical space, which refers to the range of compounds that can be extracted, ionized, and detected by a given analytical method or workflow.^17^ This inherent limitation means that any analytical approach, whether targeted or nontargeted strategy, results in a limited chemical space. The development of versatile ionization methods may expand the chemical space covered in these multiclass assays and gather meaningful information from a single acquisition run/data file.

Plasma-based ionization methods have emerged as powerful alternatives to conventional ionization sources in mass spectrometry, offering multiple advantages in sensitivity, versatility, and applicability.^18^ According to the plasma formation approaches, these ion sources can be further classified into corona discharge techniques, glow discharge techniques, microwave-induced plasma techniques, and dielectric barrier discharge (DBD) techniques.^19^ Among them, dielectric barrier discharge-based ionization has gained particular interest due to its remarkable versatility and broad chemical coverage, enabling efficient ionization of both polar and nonpolar species.^20^ The DBD technique involves the application of a high-voltage alternating current (AC) between two electrodes separated by a dielectric layer, typically using a noble gas to generate an electric discharge. Several DBD-based ion sources have been developed, with some of the most representative being low-temperature plasma (LTP),^21^ dielectric barrier discharge ionization (DBDI),^22^ active capillary plasma ionization (ACaPI)^23^ and inverse-voltage LTP (i-LTP).^24^ The applicability of DBD has been reported in fields ranging from environmental science,^25^ food safety,^26^ and biological analysis,^27^ exhibiting good performance in both ambient ionization format,^28^ as well as in combination with separation techniques like LC–MS,^29^ GC–MS,^30^ or ion mobility spectrometry (IMS).^31^ Flexible microtube plasma (FμTP)^32^ can be somewhat regarded as a dielectric guided discharge that resembles these ionization methods. Yet, it has a singular electrode architecture as it does not include a second grounded electrode, between the HV electrode and the dielectric material. This allows some beneficial features in terms of footprint, lower power, and discharge consumption as well as the simple miniaturization of ionization devices.^32^

It is commonly assumed that the soft ionization mechanism of plasma-based ion sources resembles the APCI reactions involving water cluster formation. Nevertheless, recent studies have revealed differences in the ionization mechanisms associated with APCI and DBDI when analyzing vaporized liquid samples.^33^ This suggests that the nature of the discharge can be influenced by several factors, including the discharge gas used. Helium is commonly used in soft ionization plasma sources due to the high energy of the metastable helium (He^M^) atoms produced during the discharge. These atoms can ionize N_2_ through Penning ionization, usually leading to the formation of both the molecular ion and the protonated molecule.^34^ As for argon plasmas, the ionization mechanism responsible for the formation of [M + H]^+^ and [M]^+·^ is not yet fully elucidated since argon metastable (Ar^M^) atoms lack sufficient energy to induce Penning ionization of N_2_. Some studies have suggested that the Ar^+^ and Ar_2_^+^ ions generated in the argon plasma might have enough energy to ionize water molecules or react with the analyte by charge transfer.^35^ Moreover, the presence of trace impurities in the discharge gas has been shown to influence ionization. Specifically, the argon-propane mixture has demonstrated similar behavior to that observed in He and N_2_ systems, as propane can undergo Penning ionization by argon metastable atoms.^36,37^ Focusing on the FμTP source, a recent study examined the distinct discharge behaviors of helium and argon gases. It was found that N_2_^+^ ions primarily maintain the plasma in the He–FμTP system, whereas Ar^+^ ions are responsible in the Ar–FμTP system. In contrast, for the Ar-propane-FμTP system, propane ions are the main drivers of plasma generation.^38^

As for the nature of the discharge gas, according to the use and existing literature, and with a few exceptions^39,40^ helium is the preferred or more common, considering the higher energy carried by the relatively long-lived He^M^.^41^ However, helium is not ideal for the turbopumps of mass spectrometers, and its use at high flow rates can lead to device shutdown.^36^ Additionally, the decline in natural helium deposits presents another concern. As a result, alternative discharge gases, such as argon, are being increasingly employed. Argon can be extracted from the air and does not cause issues in the vacuum system of the mass spectrometer.^39^ However, despite the increasing number of studies based on argon plasmas,^34,38^ the core ionization mechanism remains challenging and not fully understood. Since the FμTP were operated not only with He and Ar but also with Kr and Xe and similar ionization efficiencies were measured, it can be excluded that Penning ionization or charge transfer between components of the plasma gas and the surrounding air can be the dominant ionization mechanisms.^34,38,42–44^

The present study aimed to evaluate the usefulness of FμTP as an ionization source for LC–MS analysis of a broad range of pesticides, including ESI-amenable and organochlorine pesticides and other related chlorinated contaminants. The analytical performance of FμTP was compared with commercial ESI and APCI ionization sources in terms of sensitivity, analyte coverage, and tolerance to matrix effects. Additionally, this study explored the use of several discharge gases for FμTP ionization, including helium, argon, and argon-propane. The main objectives were: (i) the assessment of novel discharge gases and their performance for FμTP ionization; (ii) test and showcase the chemical space covered by the miniaturized plasma source for a wide range of organic contaminants, including those nonamenable solely by standard LC–MS approaches.

## Experimental Section

### Chemicals and Reagents

Individual pesticide analytical standards (purity ≥ 98%) were acquired from Sigma-Aldrich (Steinheim, Germany). HPLC-grade solvents methanol, acetonitrile, and water were supplied by Merck (Darmstadt, Germany). Magnesium sulfate anhydrous, sodium chloride, and formic acid were also purchased from Sigma-Aldrich (Steinheim, Germany). Primary-secondary amine (PSA) and the sorbent Enhanced Matrix Removal-Lipid (EMR) were obtained from Agilent Technologies (Santa Clara, CA, USA). Individual pesticide solutions (ca. 500 mg L^–1^ each) were prepared in acetonitrile and stored at −20 °C. Working solutions were prepared by appropriate dilution of the stock solutions with methanol and water to match the initial mobile phase composition of the gradient elution method. Helium (99.9999% purity), argon (99.999% purity), and a gas mixture of argon (99.999% purity) containing 3000 ppm of propane (Air Liquide, Spain) were evaluated as discharge gases.

### Sample Treatment

Apple, grape, and avocado were selected as representative matrices due to their classification within distinct matrix groups established in the SANTE Guidance Document on Pesticide Analytical Methods.^45^ This document categorizes food matrices based on their predominant compositional characteristics: high water content (apple), high acid content (grape) and high oil content (avocado). All extracts were obtained by the QuEChERS method,^46^ following the same general procedure. For apple and grape, 10 g of homogenized sample were weight directly into a 50 mL centrifuge tube. For avocado, due to its low water content, 3 g of homogenized sample were combined with 7 mL of water to reach a consistent extraction mass of 10 g. In all cases, the sample was mixed with 10 mL of acetonitrile and vigorously shaken for 1 min. Then, 4 g of MgSO_4_ anhydrous and 1 g of NaCl were added, and the tube was immediately shaken for 1 min. The extract was centrifuged at 3500 rpm (1300*g*) for 5 min.

The cleanup step was subsequently performed on the resulting extracts and differed according to matrix type. For apple and grape (nonfatty matrices), a 5 mL aliquot of the supernatant was transferred to a 15 mL centrifuge tube containing 250 mg PSA and 750 mg MgSO_4_ anhydrous. The mixture was shaken for 30 s and centrifuged at 3500 rpm (1300*g*) for 5 min. Subsequently, the extract was filtered through a PTFE filter (0.45 μm) and subjected to a 1:5 dilution with the initial composition of the LC mobile phase. For avocado (fatty matrix), cleanup was carried out using EMR sorbent: 1 g of EMR sorbent was activated with 5 mL of water before use. Then, 5 mL of acetonitrile extract from the sample partitioning was added, and the tube was shaken for 1 min and centrifuged at 3500 rpm (1300 x *g*) for 5 min. After this step, 5 mL of the supernatant obtained was transferred to a second centrifuge tube, containing 1.6 g MgSO_4_ and 0.4 g NaCl, shaken and centrifuged. Finally, the extract was filtered through a PTFE filter (0.45 μm) and subjected to a 1:5 dilution with the initial composition of the LC mobile phase.

### Liquid Chromatography/Mass Spectrometry (LC–MS)

An ultrahigh-performance liquid chromatograph (UHPLC) Dionex Ultimate 3000 (Thermo Fisher Scientific, Waltham, MA, USA) was used with an Agilent Zorbax Eclipse Plus C18 column (100 mm × 2.1 mm, 1.8 μm) (Agilent Technologies, Santa Clara, CA, USA). The mobile phase consisted of water (A) and methanol:water (95:5) (v/v) (B), both with 0.1% formic acid for ESI-amenable pesticides and without the organic acid for organochlorine compounds. The gradient started at 10% B. After 3 min, a linear gradient up to 100% B was set for 15 min and then held constant for 2 min. The mobile phase returned to the initial composition at 20 min. After each run, a 3 min postrun equilibration was performed with the initial mobile phase composition. The elution gradient for organochlorine pesticides started at 70% B with a linear gradient reaching 100% B at 5 min. This composition was held constant for 5 min and the mobile phase returned to the initial composition at 10 min. A 3 min postrun equilibration was performed with the initial mobile phase composition. The flow rate was set at 0.3 mL min^–1^ and the injection volume was 10 μL.

The UHPLC system was coupled to a TSQ Quantiva triple quadrupole analyzer (Thermo Fisher Scientific, San José, CA, USA) equipped with an Ion Max NG API ionization source, which can operate with either heated electrospray ionization (H-ESI) or APCI sources. For the analysis of ESI-amenable pesticides (positive ionization mode), it operated in Multiple Reaction Monitoring (MRM) acquisition mode (Q1 and Q3 resolution: 0.7 FWHM), detecting the protonated ion [M + H]^+^ as precursor ion for all compounds. Specific MRM parameters, including quantifier (*Q*) and qualifier (*q*) ions, are detailed in Table S1 (Supporting Information (SI)). For organochlorine compounds (negative ionization mode), the full-scan mode was used across the range *m*/*z* 50–600 (Q1 resolution: 0.7 FWHM), due to the high stability of these compounds resulting in low sensitivity of the MRM transitions. Instrumental parameters for both positive and negative ionization modes were optimized according to the requirements of the respective ionization sources (Table 1). Chromeleon CDS software (version 6.8) and TSQ Quantiva Tune software (version 2.1) were used to control the UHPLC system and acquire the MS data. Qualitative and quantitative data visualization were performed using Xcalibur 3.0 software (Thermo Fisher Scientific).

**Table 1:** Optimized Conditions for the Different Ionization Sources Evaluated^a^

**parameter**	**ESI**	**APCI**	FμTP
sheath gas (Arb)	50	45	45
auxiliary gas (Arb)	15	5	5
sweep gas (Arb)	0	0	0
ion transfer tube temp.	350	275	275
vaporizer temperature (°C)	400	350	350
spray voltage (KV) (±)	3.5/2.5	N/A	N/A
corona current (μA)	N/A	4(+)/10(−)	N/A
discharge gas flow rate (mL min^–1^)	N/A	N/A	50
AC voltage amplitude (kV)	N/A	N/A	2.2

a N/A: Not applicable.

### Ionization Sources: FμTP, APCI, and ESI

The FμTP source was implemented into the commercial API source housing (Thermo Fisher Scientific, San Jose, CA, USA) with an orthogonal configuration relative to the vaporizer and the mass spectrometer inlet (Figure 1B). The vaporization of the LC eluent in FμTP occurred in the same manner as in APCI, with the main difference being the absence of the corona discharge needle in the FμTP setup. The FμTP source consists of a flexible polyimide-coated fused silica capillary (ID = 250 μm, OD = 350 μm) fitted into a polyether ether ketone (PEEK) T-piece, which enables the insertion of the pin electrode (flexible tungsten wire) into the capillary. The tungsten wire (OD = 100 μm) is used as a high-voltage electrode, with no ground electrode being necessary for this discharge as the inner surface of the capillary acts as grounded electrode (Figure 1A).^32^ An in-house made square-wave generator supplies the plasma operating voltage, with a maximum voltage of 3.5 kV at a frequency of 20 kHz and rates of voltage rise of 60 V ns^–1^. Helium (99.9999% purity), argon (99.999% purity), and a gas mixture of argon (99.999% purity) containing 3000 ppm of propane (Air Liquide, Madrid, Spain) were evaluated as discharge gases. Several ionization conditions were optimized for FμTP (Figure S1), being the most influential parameters the AC voltage amplitude and the discharge-flow rate. Optimal conditions were established as 2.2 kV for AC voltage amplitude and 50 mL min^–1^ for discharge-flow rate. A summary of the remaining optimized FμTP parameters, along with those for ESI and APCI, is provided in Table 1.

**Figure 1:**
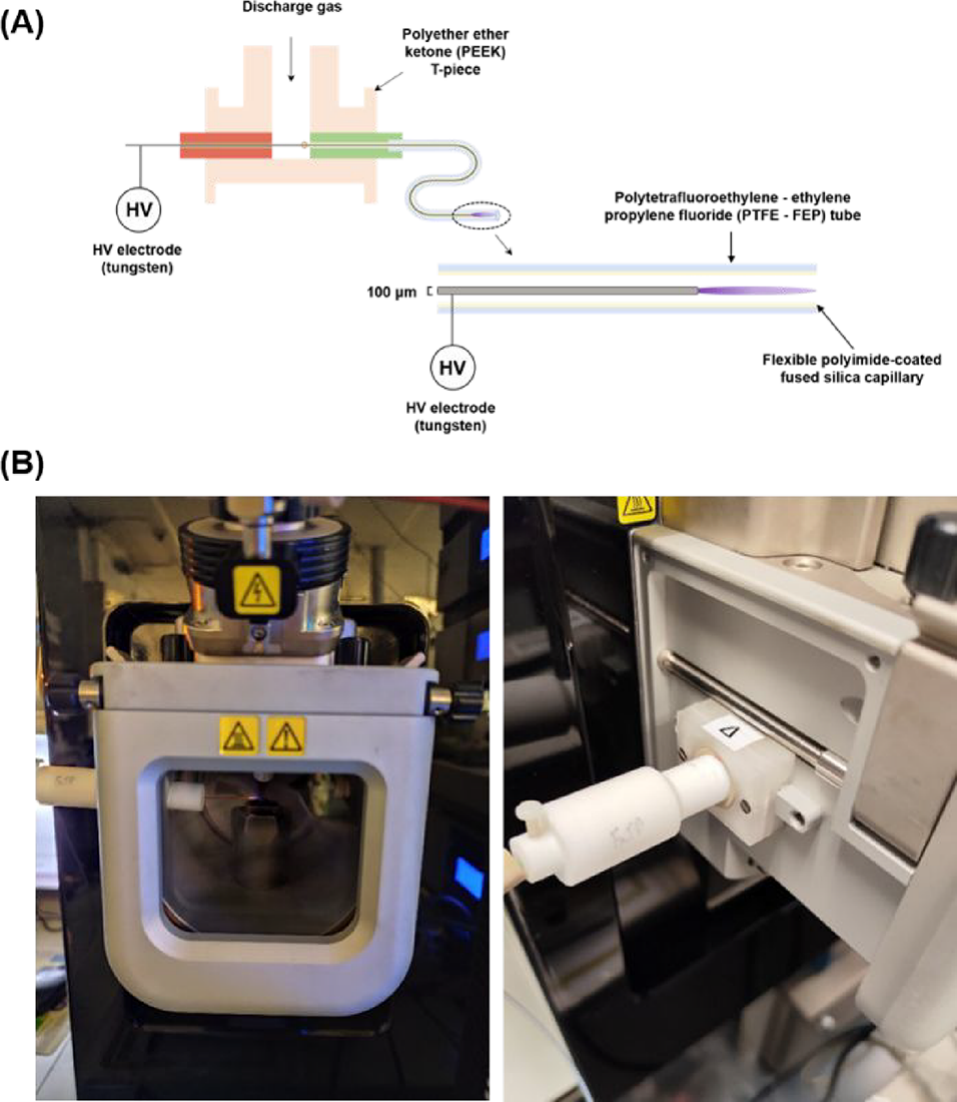
(A) Schematic illustration of the FμTP source. (B) Setup of the FμTP ionization source coupled into the commercial API source housing.

## Results and Discussion

### Effect of Discharge Gases on the Ionization Pathways of FμTP Source

Detailed information on the main mass spectral features of the multiclass pesticides analyzed in positive ion mode using ESI, APCI, and FμTP (with different discharge gases) is provided in Table S2. Data includes full-scan spectra for each ionization source. All the evaluated sources exhibited soft ionization behavior, as evidenced by minimal fragmentation. Similar spectra were observed across ESI, APCI, and FμTP in positive mode analysis, with the protonated ion [M + H]^+^ consistently detected for all pesticides. The main difference between these three ionization sources was the absence of sodium adducts with APCI and FμTP, regardless of the discharge gas used in the latter sources (Figure 2). This phenomenon may be attributed to the fact that the ionization occurs in the gas phase, in contrast to ESI, suggesting that gas-phase reactions could minimize/hinder adduct formation. Sodium adduct formation is a well-known issue in ESI, often complicating the interpretation of spectra (especially in MS/MS analysis) and leading to reduced sensitivity by distributing the analyte signal among multiple ions.^6^ Furthermore, sodium adducts have been shown to enhance matrix effects, with their formation in ESI being significantly influenced by the concentration of inorganic ions present in the sample.^47^ While sodiated ions can sometimes provide intense signals that may be advantageous for certain compounds, their presence generally adds complexity to spectral interpretation and may affect the robustness of quantitative measurements. Additional examples showing the reduction of sodium adducts in APCI and FμTP are shown in Figure S2. In this context, the plasma-based ionization source proposed in this study offers a significant advantage over electrospray ionization, as FμTP has shown the ability to suppress the adduct formation due to the different ionization pathways.

**Figure 2:**
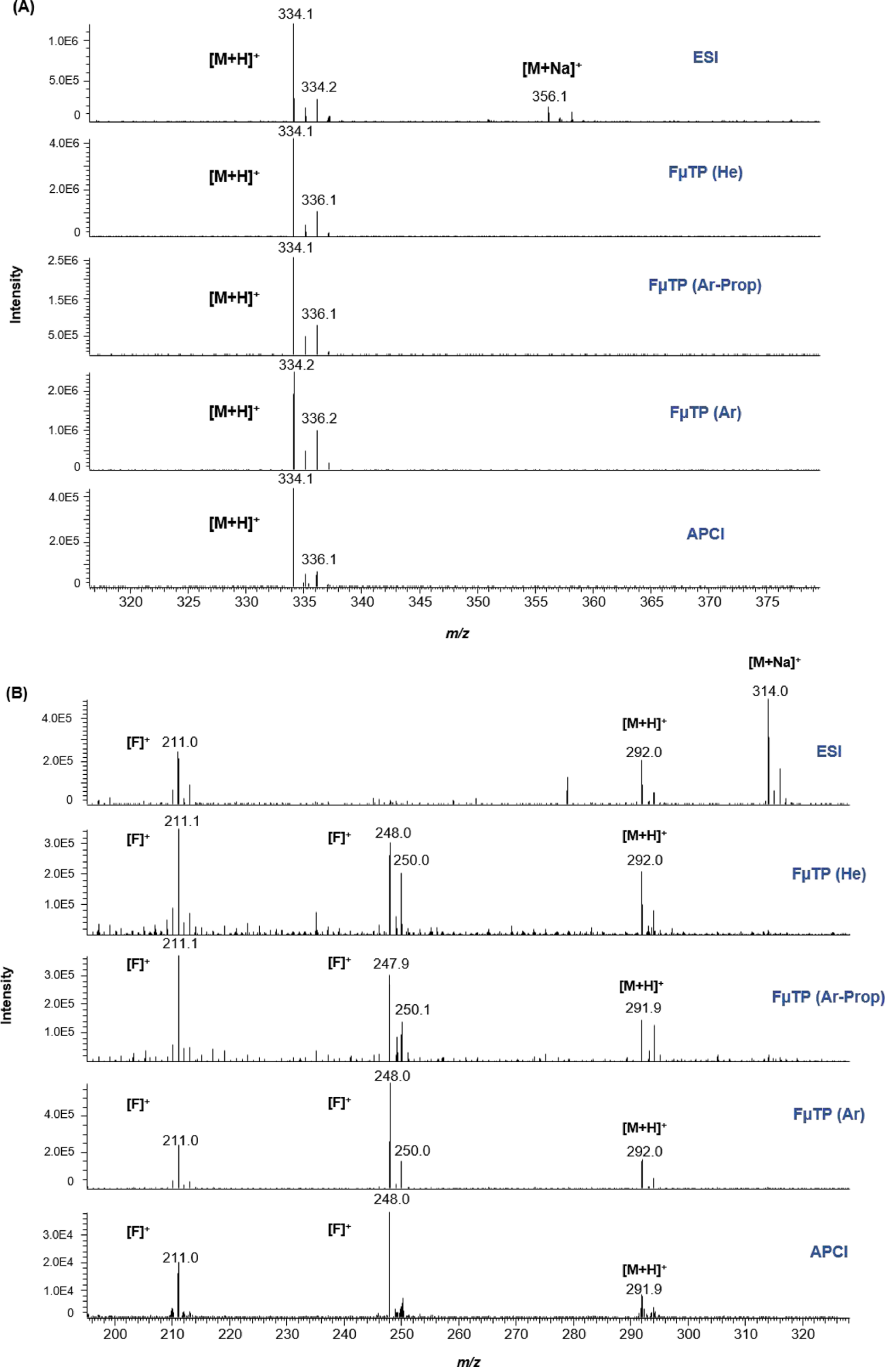
Mass spectral features for (A) tebufenpyrad and (B) thiamethoxam using ESI, FμTP (helium, argon-propane, argon) and APCI. Spectra acquired in full scan mode at a concentration level of 50 pg μL^–1^. The ion [F]^+^ is referred to as a fragment. For details, see text.

An example to illustrate the differences of the three tested sources in the positive ionization mode is the case of thiamethoxam (Figure 2B). The ESI mass spectrum revealed that the [M + Na]^+^ ion was the most abundant, exceeding both the [M + H]^+^ ion and the fragment at *m*/*z* 211. In contrast, APCI and FμTP not only reduced the formation of the sodium adduct but also detected a highly sensitive ion at *m*/*z* 248. This ion is attributed to a typical degradation product of neonicotinoid pesticides, likely generated in the APCI source via in-source ion/molecule reactions involving water molecules.^48^ Analogous mass spectra were observed across all FμTP systems evaluated (Table S2).

In contrast, more significant variations were observed in negative ionization mode. All organochlorine compounds were evaluated using ESI, APCI and FμTP, including the use of different discharge gases in the miniaturized plasma source. Detailed information regarding the mass spectral features obtained with each ionization source is summarized in Table 2. The results showed that ESI was only capable of ionizing chlorothalonil and endosulfan sulfate, while the remaining pesticides were not detected under these conditions even at high concentrations (up to 10 mg L^–1^). These findings are consistent with previous studies based on the analysis of organochlorine compounds using ESI ionization, further corroborating its limited effectiveness for this class of pesticides.^12^ In negative ion mode, a diverse range of reagent ions such as O_2_^–·^, NO_2_^–^, and NO_3_^–^, are mainly responsible for ionizing the analyte in plasma-based sources, leading to a wide variety of product ions generated.^49^ Notably, organochlorine pesticides with aromatic structures exhibit high chemical stability due to the presence of π-electron conjugation and strong carbon-chlorine (C–Cl) bonds.^50^ This stability resulted in relatively low sensitivity for most of the pesticides analyzed in both MRM transitions, so the Q1 full scan mode was used for detection. Chlorothalonil, pentachlorobenzene, hexachlorobenzene, quintozene, and chlorpyrifos ethyl exhibited [M–Cl+O]^−^ ions obtained from nucleophilic aromatic substitution reactions.^51^

**Table 2:** Mass Spectral Features of Organochlorine Contaminants with FμTP, APCI, and ESI^b^

**compound**	***R_t_*** (min)	**formula**	**ionization source**	***m*/*z***	**detected ions**
captan^a^	2.50	C_9_H_8_Cl_3_NO_2_S	FμTP (He, Ar-Prop, Ar); APCI	150.1	**[M–CSCl**_**3**_ **]** ^**–**^
folpet^a^	3.00	C_9_H_4_Cl_3_NO_2_S	FμTP (He, Ar-Prop, Ar); APCI	146.1	**[M–CSCl**_**3**_ **]** ^**–**^
chlorothalonil	3.13	C_8_Cl_4_N_2_	FμTP (He, Ar-Prop, Ar); APCI; ESI	244.9	**[M–Cl+O]** ^**–**^
captafol^a^	3.17	C_10_H_9_Cl_4_NO_2_S	FμTP (He, Ar-Prop, Ar); APCI	150.1	**[M–SC**_**2**_ **Cl**_**4**_ **]** ^**–**^
endosulfan sulfate	3.96	C_9_H_6_Cl_6_O_4_S	FμTP (He, Ar-Prop, Ar); APCI	384.9	**[M-Cl]** ^**–**^
				418.8	[M–H]^−^
			ESI	418.8	**[M–H]** ^**–**^
β-endosulfan^a^	4.96	C_9_H_6_Cl_6_O_3_S	FμTP (He)	267.0	fragment
				302.9	fragment
				338.9	[M–HSO_2_]^−^
				368.8	[M–Cl]^−^
				402.8	**[M–H]** ^**–**^
			FμTP (Ar-Prop, Ar)	229.0	fragment
				267.0	fragment
				302.9	**fragment**
			APCI	302.9	fragment
				368.8	[M–Cl]^−^
				402.8	**[M–H]** ^**–**^
chlorpyrifos ethyl^a^	5.38	C_9_H_11_Cl_3_NO_3_PS	FμTP (He, Ar-Prop, Ar)	312.9	[M–H-Cl]^−^
				329.9	**[M–Cl+O]** ^**–**^
			APCI	312.9	**[M–H-Cl]** ^**–**^
α-endosulfan^a^	5.52	C_9_H_6_Cl_6_O_3_S	FμTP (He)	229.0	fragment
				267.0	fragment
				302.9	fragment
				338.9	**[M–HSO**_**2**_ **]** ^**–**^
				384.8	[M–Cl+O]^−^
				402.8	[M–H]^−^
			FμTP (Ar-Prop, Ar)	229.0	**fragment**
				267.0	fragment
				302.9	fragment
			APCI	229.0	**fragment**
				267.0	fragment
				402.8	[M–H]^−^
dicofol^a^	5.67	C_14_H_9_Cl_5_O	FμTP (He, Ar-Prop, Ar); APCI	263.1	**[M–Cl**_**3**_ **]** ^**–**^
quintozene^a^	5.84	C_6_Cl_5_NO_2_	FμTP (He, Ar-Prop, Ar)	262.9	[M–NO]^−^
				273.8	**[M–Cl+O]** ^**–**^
			APCI	246.8	[M–NO_2_]^−^
				262.9	**[M–NO]** ^**–**^
				273.8	[M–Cl+O]^−^
pentachlorobenzene^a^	6.51	C_6_HCl_5_	FμTP (He, Ar-Prop, Ar); APCI	228.9	**[M–Cl+O]** ^**–**^
hexachlorobenzene^a^	7.62	C_6_Cl_6_	FμTP (He, Ar-Prop, Ar); APCI	262.8	**[M–Cl+O]** ^**–**^

a Not detected by ESI.

b Spectra acquired in full scan mode at a concentration level of 50 pg μL^–1^ in solvent. The most abundant ion detected under these conditions is shown in bold. Only endosulfan sulfate and chlorotalonil were detected with ESI.

Slight variations in the relative abundances of ions generated by APCI and FμTP were observed for quintozene and chlorpyrifos ethyl (Figure S3). In FμTP systems, [M–Cl+O]^−^ was the predominant ion for both pesticides. In contrast, the [M–NO]^−^ ion was detected as the most abundant species for quintozene using the APCI source, which also generated the [M–NO_2_]^−^ ion. Meanwhile, for chlorpyrifos ethyl only the [M–H–Cl]^−^ ion was detected with APCI. Furthermore, some phthalimide-related compounds such as dicofol, folpet, captan and captafol were also evaluated. Similar spectra were obtained for these compounds across the different sources studied, detecting the species [M–Cl_3_]^−^ for dicofol, [M–CSCl_3_]^−^ for captan and folpet, and [M-SC_2_Cl_4_]^−^ for captafol. Each of these fragments presented an *m*/*z* corresponding to the deprotonated molecule of its phthalimidic metabolite, suggesting a source fragmentation similar to the naturally occurring degradation of these compounds.^52^

Further pesticides for which notable differences between ionization sources and discharge gases were observed include endosulfan sulfate, α-endosulfan and β-endosulfan. Endosulfan sulfate was one of the two organochlorine compounds detectable by ESI, where only the deprotonated [M–H]^−^ molecule was observed. In contrast, FμTP and APCI yielded different results, with the primary ion detected corresponding to the loss of a chlorine atom from the molecule [M–Cl]^−^, while the [M–H]^−^ ion appeared with lower relative abundance compared to ESI (Figure S4A). The presence of these species in plasma-based ionization sources can be explained by electron capture reactions, which lead to the formation of [M–Cl]^−^, or by proton abstraction mechanisms that generate [M-H]^−^ ions, both involving the peroxide radical anion (O_2_^–·^).^49^ Regarding the use of different discharge gases in FμTP, no significant differences were observed for endosulfan sulfate when using helium, argon, or argon-propane.

However, the discharge gas had a relevant impact on the ionization of α and β endosulfan isomers. For β-endosulfan, five different species were detected in FμTP using helium as discharge gas. Among these, the [M–H]^−^ ion was the predominant ion, detecting other species such as [M–Cl]^−^, [M–HSO_2_]^−^, and two additional fragments at *m*/*z* 267.0 and 302.9. Nevertheless, significant differences were observed when using argon and argon-propane as discharge gases, due to the [M–H]^−^, [M–Cl]^−^, [M–HSO_2_]^−^ ions were not detected under these conditions (Figure 3). A fragment at *m*/*z* 229 was observed with argon and argon-propane instead. A similar ionization pattern was noted for α-endosulfan, where the use of argon and argon-propane again did not yield selected ions, which were only generated when using helium (Figure S4B). These differences between helium, argon, and argon-propane could be attributed to the distinct ionization mechanisms associated with each gas. Overall, a general comparison of the results obtained for ESI-amenable and organochlorine pesticides emphasizes the substantial differences between the mechanisms involved in positive and negative ionization modes.

**Figure 3:**
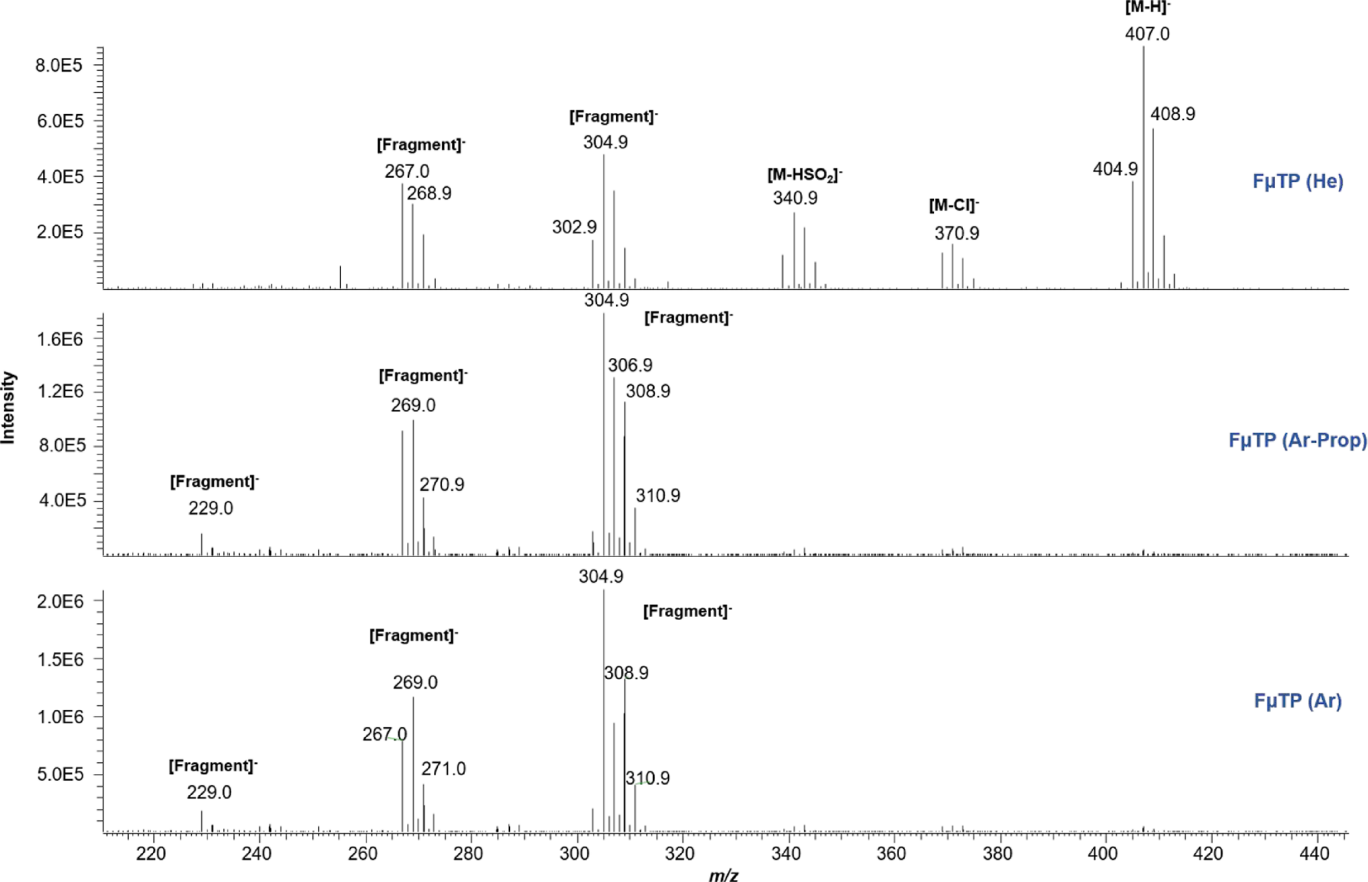
Mass spectral features for β-endosulfan using FμTP with helium, argon-propane, and argon as discharge gases. Spectra acquired in full scan mode at a concentration level of 5 mg L^–1^. For details, see text.

### Analytical Performance of FμTP for Pesticide Analysis

Several analytical parameters were evaluated for ESI, APCI, and FμTP ionization sources, including linearity, limits of quantification (LOQs), and matrix effects. Calibration curves were prepared using both solvent and matrix-matched standards, ranging from 0.05 to 50 μg L^–1^ for positive ionization mode and from 5 to 500 μg L^–1^ for negative ionization mode. Good linearity was obtained for all pesticides, with regression coefficients above 0.99. The LOQs were established according to the minimum analyte concentration yielding a signal-to-noise ratio (S/N) = 10, using the less abundant (confirmatory) MS/MS transition for each compound.

A comparison of LOQ values obtained with FμTP, ESI, and APCI (Table S3) shows that FμTP achieves comparable sensitivity to ESI for most compounds, with LOQs in a similar range. Although FμTP often provided higher signal intensities, the increased baseline noise (probably caused by additional chemical background from the plasma) reduced overall sensitivity improvements, which may explain why higher signals did not always lead to lower LOQs. In a few specific cases, such as for rotenone or imidacloprid, FμTP demonstrated slightly improved LOQs. While the differences are not always pronounced, these results suggest that FμTP can offer competitive performance. This highlights its potential as a versatile alternative to conventional ionization sources in multiclass pesticide analysis. Moreover, the use of alternative plasma gases such as argon and argon–propane mixtures was also investigated. This choice was motivated by their promising behavior in previous DBDI studies using a 2-ring electrode configuration, as reported by Schütz et al.^36^ Sensitivity results comparable to those obtained with helium were achieved using both gases, with nearly 90% of the pesticides in positive mode and 80% of the organochlorine compounds showing similar LOQs to those obtained with helium. Although their performance in the present FμTP setup did not exceed that of helium, they present practical advantages in terms of cost and availability.

#### Precision

Additionally, intraday and interday precision were assessed for the FμTP system with He, yielding an average relative standard deviation (RSD) for intraday reproducibility (*n* = 6) of 3.9% for ESI-amenable pesticides and 7.1% for organochlorine contaminants. Interday RSD values (*n* = 5) averaged 7.2 and 11.1% for positive and negative ionization modes, respectively. Data obtained for these experiments are shown in Tables S4 and S5. The quality parameter ranges were in the same range as the specifications claimed by the commercial vendor source.

#### Sensitivity

A source comparison study was performed to evaluate the sensitivity offered by ESI, APCI, and FμTP for pesticide analysis. In positive ionization mode, FμTP exhibited better performance than ESI and APCI for a significant fraction of the compounds analyzed. Figure 4A shows the extracted ion chromatograms (EIC) obtained from selected pesticides using FμTP, ESI and APCI, highlighting the enhanced performance of FμTP. This increase in signal intensity can be partially attributed to the reduction of sodium adduct formation, as previously discussed. Furthermore, the sensitivity improvement provided by FμTP was also notable for pesticides ionized in negative mode, with mass spectra revealing signal enhancements of up to an order of magnitude compared to APCI (Figure S3).

**Figure 4:**
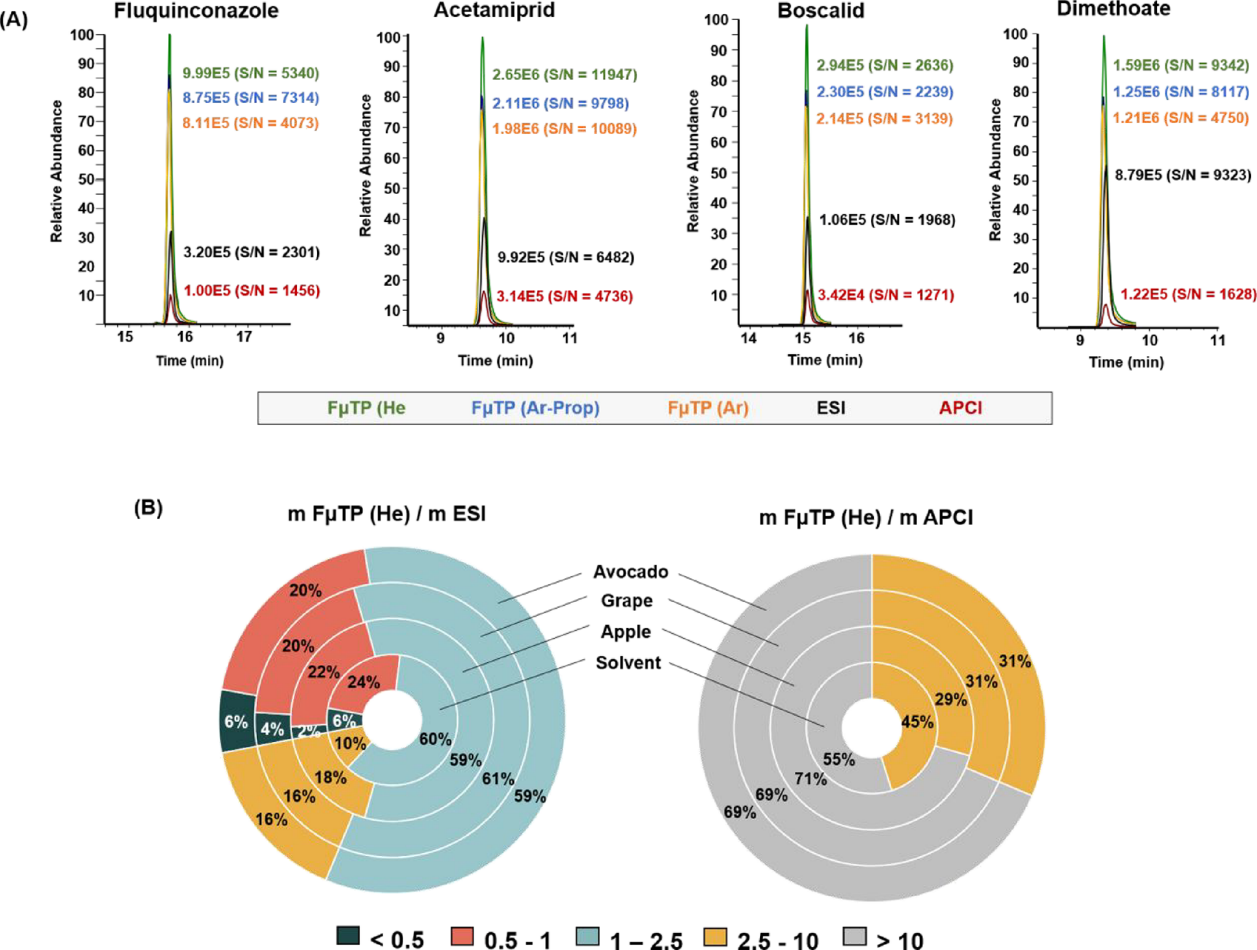
Comparison of sensitivity with ESI, APCI and FμTP for pesticides analyzed in positive ion mode by LC–MS with the different ionization methods and conditions studied. (A) Extracted ion chromatograms (EIC) for fluquinconazole, acetamiprid, boscalid and dimethoate at a concentration level of 50 μg L^–1^ in solvent. (B) Comparison of the calibration curve slopes (m) using ESI, APCI and He–FμTP in solvent and three different matrices (apple, grape, avocado). For each compound, the peak height is reported along with the signal-to-noise ratio (S/N) in brackets. For details, see text.

Additionally, a comparative study of the calibration slopes (m) obtained with ESI, APCI and FμTP was conducted using solvent-based and matrix-matched calibration curves. The results for pesticides analyzed in positive ionization mode are presented in Figure 4B. This graph illustrates the ratio of the calibration slopes for He–FμTP relative to those obtained for ESI and APCI. A slope ratio greater than 1 means higher sensitivity for FμTP, whereas values below 1 indicate superior performance for ESI or APCI. As shown in Figure 4B, 70% of the solvent-based standards exhibited a mFμTP/mESI ratio higher than 1, indicating superior sensitivity with FμTP.

This percentage increased to 75–76% when pesticides were analyzed in the different food matrices, likely due to the reduced impact of matrix effects using FμTP (discussed in detail in the following section). For the remaining compounds, 20–24% showed slightly higher signals with ESI (slope ratio between 0.5 and 1), while only 2–6% of the tested pesticides showed a clear sensitivity advantage for ESI over FμTP (slope ratio <0.5). Furthermore, comparing the slopes between FμTP and APCI revealed that FμTP was significantly more effective for ESI-amenable pesticides (Figure 4B). The percentage of compounds with a mFμTP/mAPCI ratio greater than 10 was notably higher for matrix analyses (69–71%) than solvent analyses (55%). This observation, similar to the earlier comparison between FμTP and ESI, can be attributed to the stronger matrix effects associated with the APCI source. For organochlorine contaminants, all the slopes were remarkably higher with FμTP than with APCI, as shown in Figure 5. These results were consistent across solvent and matrix-based analyses. Based on these findings, it can be concluded that FμTP generally provides good performance for pesticide analysis in both positive and negative ionization modes, enhancing analyte coverage compared to conventional sources. The sensitivity of FμTP was also evaluated as a function of the discharge gas used. For this purpose, calibration curves were compared using helium, argon, and argon-propane.

**Figure 5:**
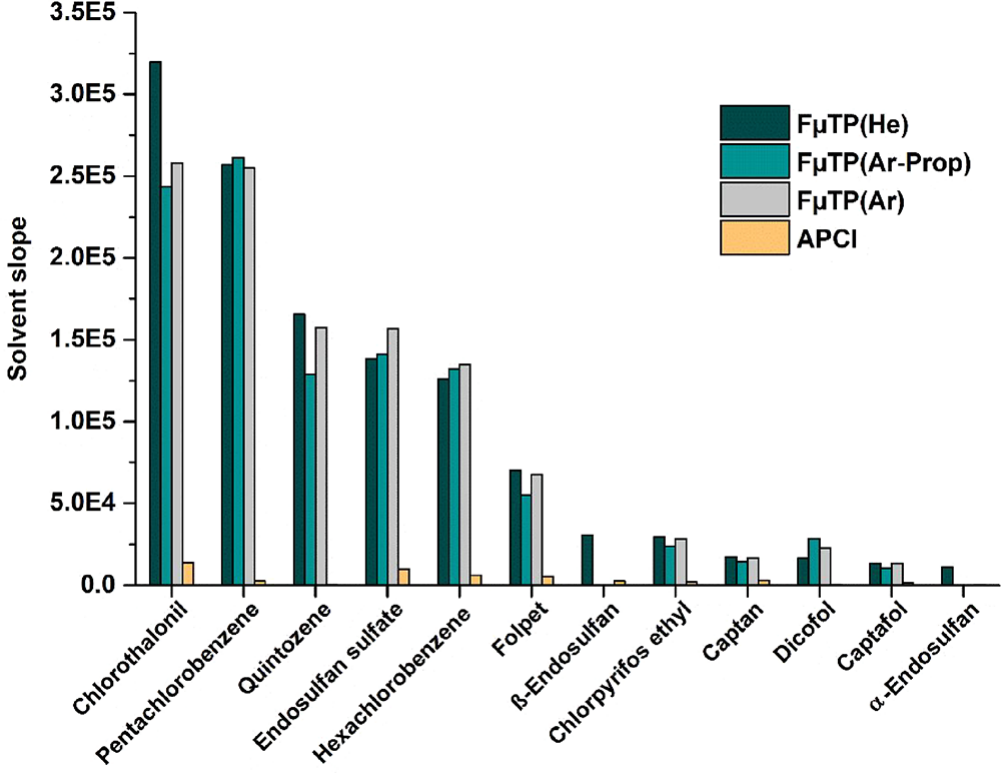
Evaluation of sensitivity with APCI and FμTP using different discharge gases for organochlorine pesticides. Comparison of the solvent calibration curve slopes.

The comparison of the slope ratios between FμTP and ESI revealed slightly lower values when using argon or argon-propane compared to helium (Figure S5). However, despite these variations, about 90% of the pesticides analyzed in positive mode exhibited LOQs similar to those obtained with helium. This comparable performance is illustrated by the chromatograms shown for several pesticides in Figure 4A. A similar trend was observed for organochlorine pesticides (Figure 5), where the calibration slopes obtained using the three discharge gases were nearly identical across all analyzed compounds except for the α- and β-endosulfan isomers, for which lower signal intensities were observed with argon and argon propane gases. This can be attributed to the different species formed with the various discharge gases, as previously discussed. These findings indicate that, in general, argon and argon-propane could be potential alternatives to helium as discharge gas for FμTP analysis without a major sacrifice in performance.

#### Matrix Effects

The present study evaluated whether FμTP reduces matrix effects compared to conventional ionization sources. Apple, grape, and avocado were selected as representative matrices due to their high water, acid, and oil content, respectively.^45^ Matrix effects were assessed following the SANTE/11312/2021 guidelines,^53^ categorizing results into four groups: negligible (≤10%), soft (10–20%), medium (20–50%), and strong (≥50%). Figure 6 shows the matrix effects for each sample with the different ionization sources, for both positive and negative ion mode targeted contaminants.

**Figure 6:**
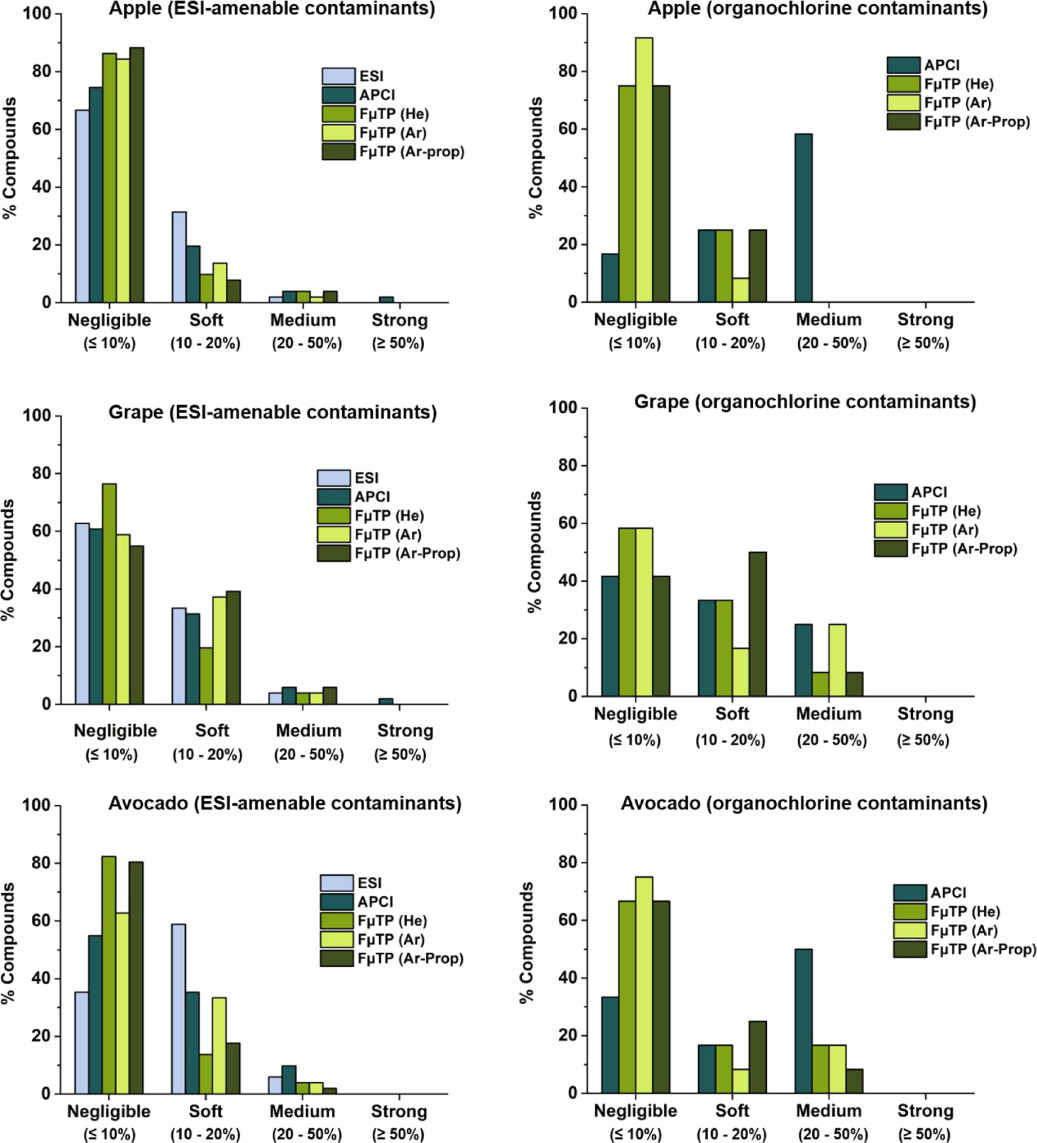
Evaluation of matrix effects in apple, grape and avocado for ESI-amenable and organochlorine pesticides using ESI, APCI and FμTP (with different discharge gases). Each graph represents the percentage of pesticides falling into each matrix effect category, classified as negligible (≤10%), soft (10–20%), medium (20–50%), and strong (≥50%) for the different ionization sources evaluated.

For ESI-amenable pesticides, the data revealed that approximately 80% of the compounds exhibited a negligible matrix effects in apple and avocado using the FμTP source. Meanwhile, the percentage of pesticides fulfilling this criterion decreased in apple to 75% with the APCI source and 67% with the ESI source. This difference between commercial and FμTP sources was more evident for avocado, where 55% of the pesticides showed negligible matrix effects for APCI and only 35% for ESI. Conversely, a similar profile was observed across all three sources for grapes, with about 95% of the pesticides showing negligible or soft matrix effects. The reduced impact of matrix effects with FμTP was more evident for organochlorine contaminants. In this case, FμTP data were compared only with APCI due to the poor performance of ESI for these compounds. In apple and avocado, approximately 70% of the pesticides yielded negligible matrix effects using FμTP, while APCI resulted in higher suppression, with about 50% of the compounds showing medium matrix effects. A similar pattern to that observed in the positive ion mode was obtained for organochlorine species on grapes, with analogous responses for APCI and FμTP. No significant differences were detected in FμTP performance with the use of different discharge gases (helium, argon, or argon-propane), further demonstrating the robustness of this source and the potential to replace helium with alternative gases in this miniaturized plasma-based system.

## Concluding Remarks

This study evaluated the performance of LC-FμTP-MS for efficient ionization of multiclass pesticides. The results offered by this miniaturized plasma-based source were compared with those obtained with conventional ESI and APCI ionization sources, showing FμTP a great potential in terms of sensitivity, reproducibility and tolerance to matrix effects. Beyond these advantages, the utility of FμTP lies in its ability to efficiently ionize both ESI-amenable pesticides and nonpolar lipophilic compounds, such as organochlorine pesticides, thereby significantly expanding the chemical coverage. A similar ionization pattern, in terms of the species generated, was observed for ESI-amenable pesticides across all three evaluated ionization sources. However, different ionization pathways were noted for organochlorine pesticides. These results emphasize the distinct ionization mechanisms involved in both positive and negative ion modes. Furthermore, this study explored the use of alternative discharge gases to helium for the FμTP source. As for the choice of discharge gases, the results demonstrated that, in general, the use of argon and argon-propane mixture provided comparable sensitivity to the helium-FμTP system while maintaining all of the previously mentioned advantages. These alternative gases were investigated to better understand their effects on plasma generation and overall system performance. Additionally, for certain organochlorine pesticides, the use of argon or an argon-propane mixture as the discharge gas resulted in the detection of different ions compared to helium, suggesting that the nature of the discharge gas may strongly influence the FμTP ionization mechanism. To sum up, FμTP emerges as a powerful alternative for the efficient ionization of a wide range of compounds with diverse physicochemical properties, expanding the chemical space covered by this method. Moreover, this approach demonstrates the flexibility and practical advantages of using alternative gases (helium, argon, argon-propane) while also enhancing the versatility and chemical information obtained from HRMS instruments operated at atmospheric pressure. Further studies are carried out to assay in environmental matrices with nontargeted approaches.

## Supplementary Material



## Notes

Additional information related to the optimization of FμTP parameters, mass spectral features for ESI-amenable and organochlorine contaminants with the different ionization sources evaluated, optimized MRM parameters, and analytical parameters evaluated for ESI, APCI, and FμTP sources (PDF)
