# Supplementary material for: Efficient and Wide Chemical-Space Ionization of Organic Contaminants Using LC–MS with a Miniaturized Plasma Source Applying Different Discharge Gases
Source: Anal Chem. 2025 Sep 16;97(38):20962–72. doi: 10.1021/acs.analchem.5c03745 (PMC12489893; doi:10.1021/acs.analchem.5c03745)
Supplement: Supplementary file 1 [file ac5c03745_si_001.pdf]

Efficient and wide chemical-space ionization of organic contaminants using LC-MS with a miniaturized plasma source applying different discharge gases

Irene Caño-Carrillo[1], Bienvenida Gilbert-López[1], David Moreno-González[1], Joachim Franzke[2]* and Juan F. García-Reyes[1]*

[1]*Analytical Chemistry Research Group, Department of Physical and Analytical Chemistry, University of Jaén, Campus Las Lagunillas, 23071 Jaén, Spain*
[2]*Leibniz Institut für Analytische Wissenschaften (ISAS e.V), 44123 Dortmund, Germany*

Table of contents

Figure S1. Optimization of the FμTP parameters including AC voltage amplitude, helium flow, vaporizer temperature, and sheath gas in positive (A) and negative (B) ion mode.

Figure S2. Mass spectral features for (A) thiacloprid and (B) rotenone using ESI, FμTP (helium, argon-propane, argon) and APCI.

Figure S3. Mass spectral features for (A) quintozene and (B) chlorpyrifos ethyl using FμTP (helium, argon-propane, argon) and APCI.

Figure S4. Mass spectra features for (A) endosulfan sulfate and (B) α-endosulfan using ESI, FμTP (helium, argon-propane, argon) and APCI.

Figure S5. Evaluation of sensitivity with ESI, APCI and FμTP using different discharge gases for ESI-amenable pesticides. Comparison of the solvent calibration curve slopes.

Table S1. Optimized MRM parameters for the determination of ESI-amenable pesticides.

Table S2. Main mass spectral features of the pesticides analyzed in positive ion mode using ESI, APCI, and FμTP (helium, argon-propane, argon).

Table S3. Limits of quantification for ESI-amenable pesticides in the different food matrices analyzed in the study

Table S4. Intra-day and inter-day precision (% RSD) for ESI-amenable pesticides using the FμTP ionization source.

Table S5. Intra-day and inter-day precision (% RSD) for organochlorine pesticides using the FμTP ionization source

***E-mail**: franzke@isas.de; jfgreyes@ujaen.es

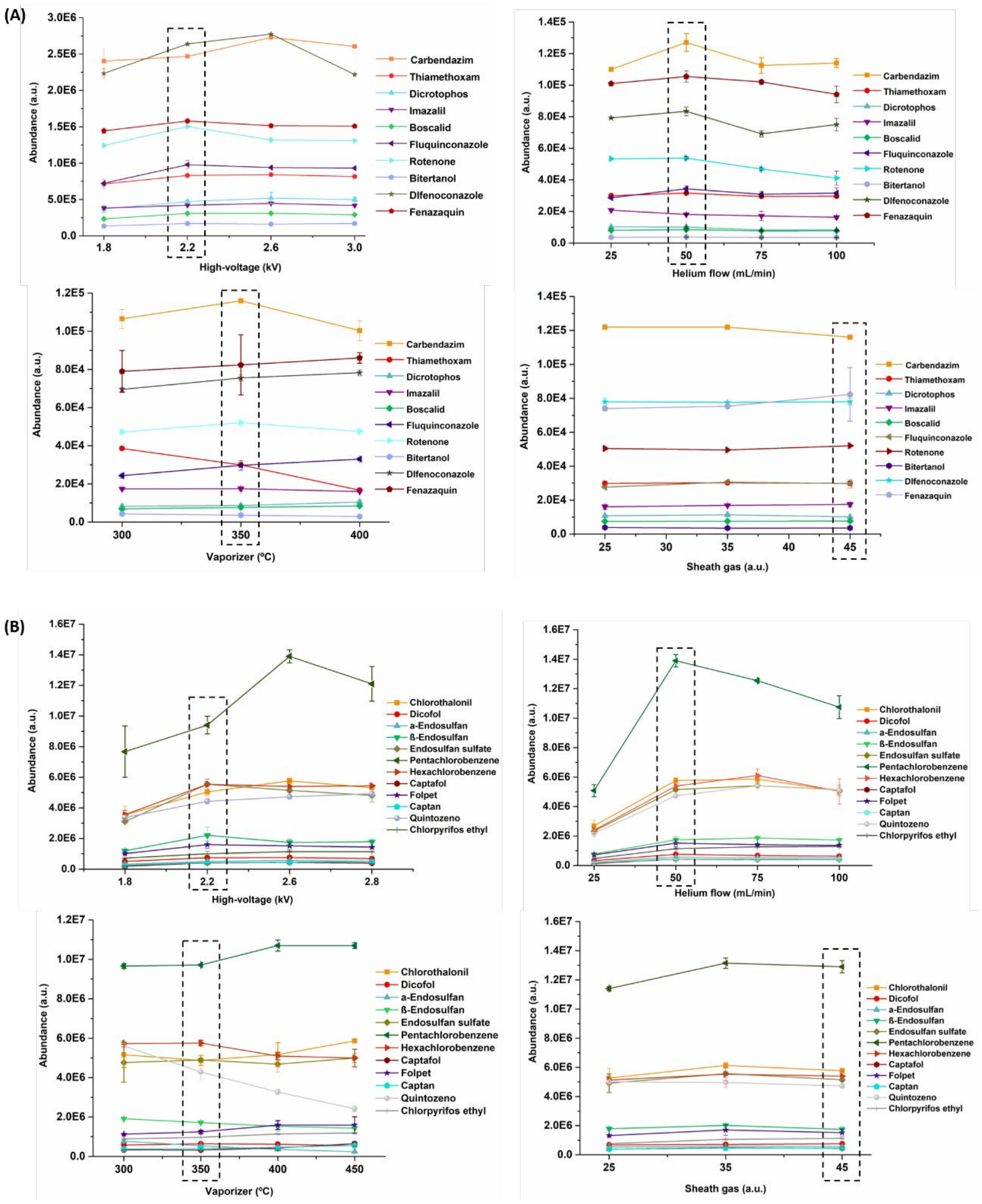

Figure S1. Optimization of the FμTP parameters including AC voltage amplitude, helium flow, vaporizer temperature, and sheath gas in positive (A) and negative (B) ion mode.

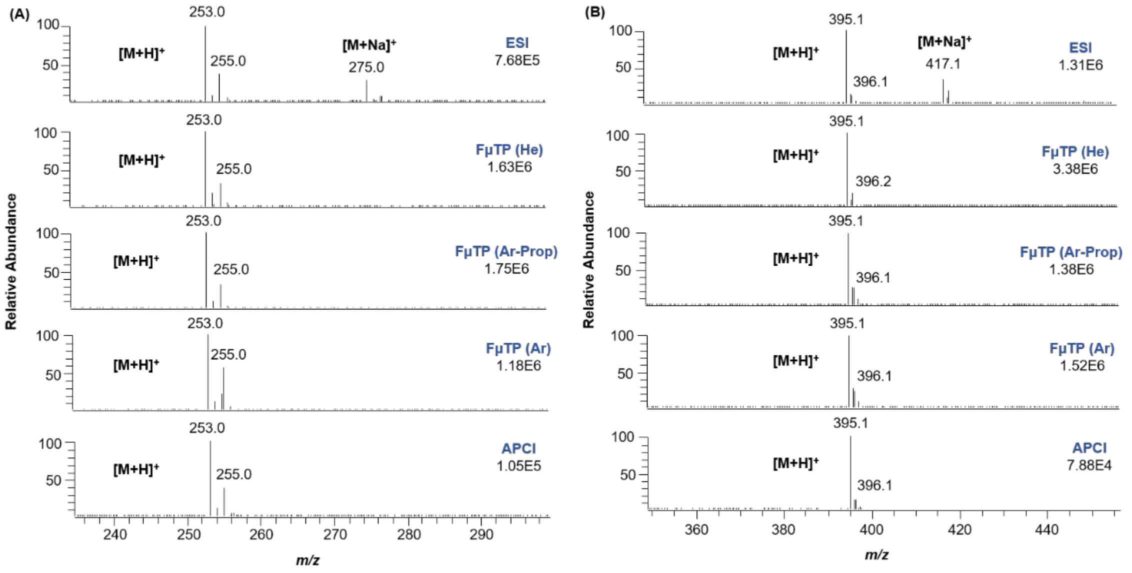

Figure S2. Mass spectra features for (A) thiacloprid and (B) rotenone using ESI, FµTP (helium, argon-propane, argon) and APCI. Spectra acquired operating in full scan mode at a concentration level of 50 µg L^{-1}. Note the sodium adduct suppression with FµTP and APCI.

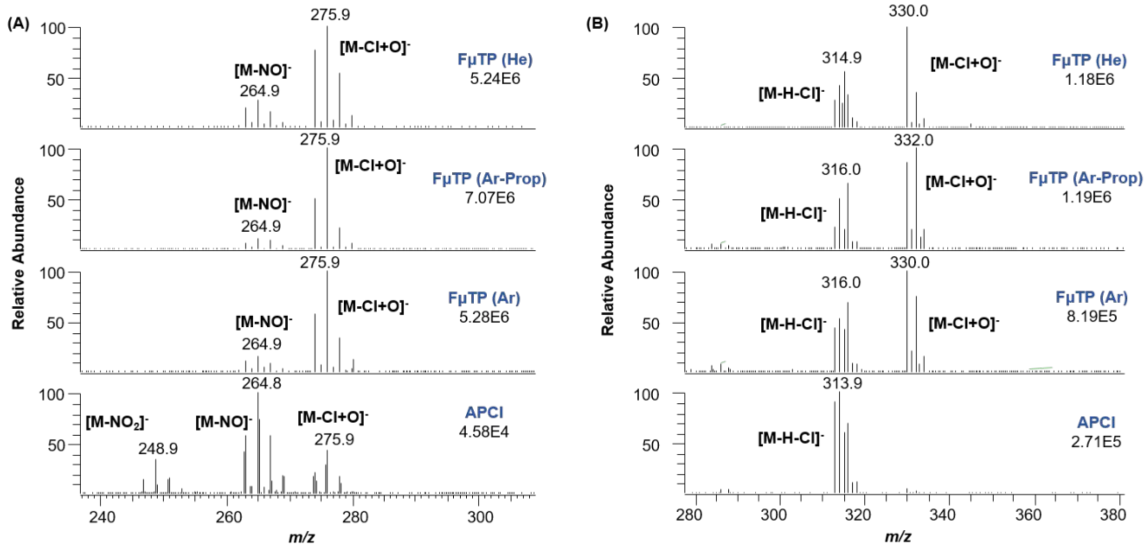

Figure S3. Mass spectra features for (A) quintozene and (B) chlorpyrifos ethyl using FµTP (helium, argon-propane, argon) and APCI. Spectra acquired operating in full scan mode at a concentration level of 500 µg L^{-1}. Note the differences in terms of ions detected between FµTP and APCI.

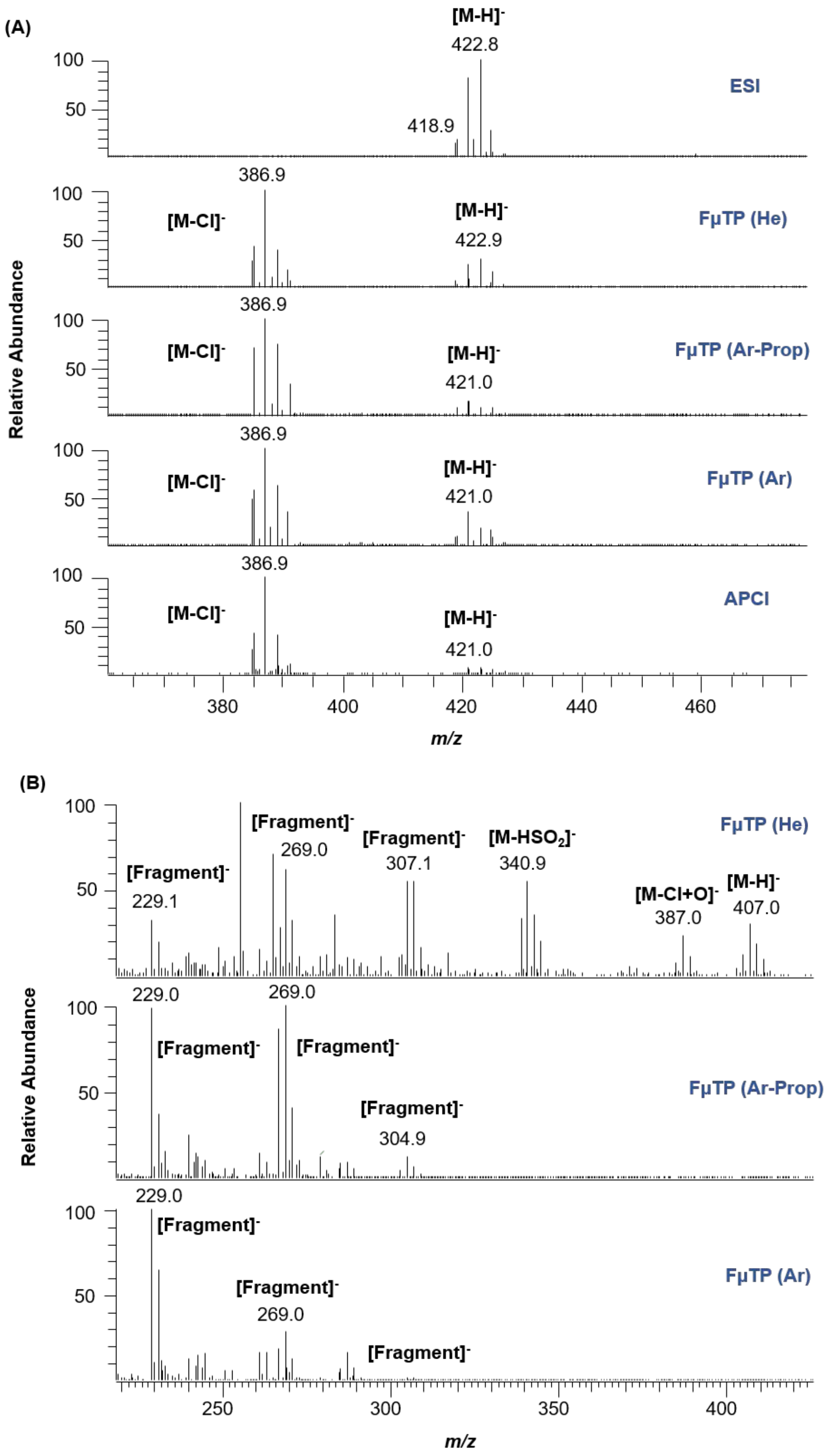

Figure S4. Mass spectra features for (A) endosulfan sulfate and (B) α-endosulfan using ESI, FμTP (helium, argon-propane, argon) and APCI. Spectra acquired operating in full scan mode at a concentration level of 500 μg L^{-1} for endosulfan sulfate and 5 mg L^{-1} for α-endosulfan. Figure A shows the differences in the ionisation of endosulfan sulphate from ESI compared to APCI and FμTP. Figure B shows that the use of argon and argon-propane as discharge gas does not lead to the formation of $[M-H]^-$, $[M-Cl]^-$, $[M-HSO_2]^-$ ions, in contrast to helium.

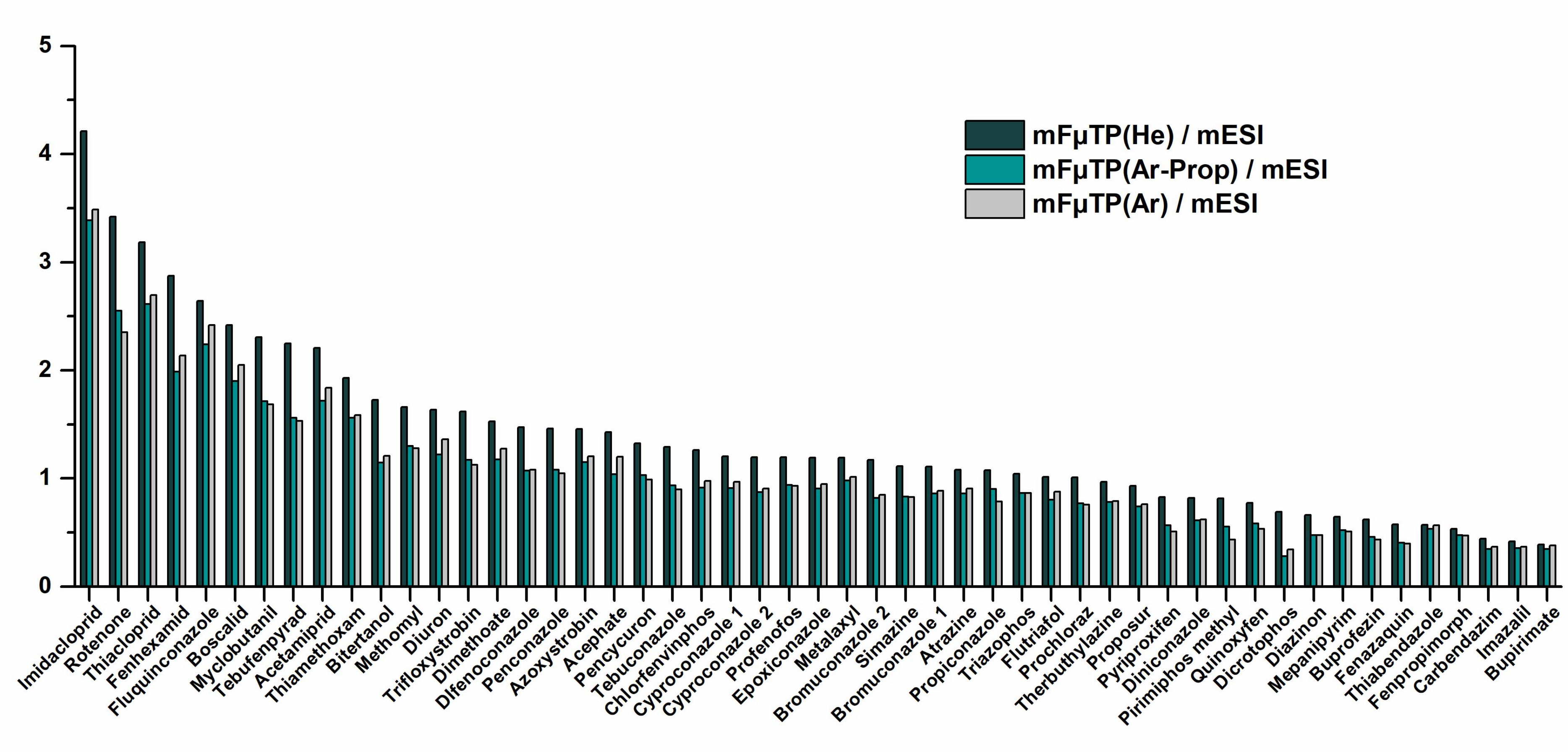

Figure S5. Evaluation of sensitivity with ESI, APCI and FμTP using different discharge gases for ESI-amenable pesticides. Comparison of the solvent calibration curve slopes.

Table S1. Optimized MRM parameters for the determination of ESI-amenable pesticides

Compound	Rt (min)	Precursor ion (*m/z*)	Quantification ion (Q) (*m/z*)	Collision Energy (V)	Confirmation ion (q) (*m/z*)	Collision Energy (V)	RF Lens (V)
Acephate	3.14	184.0	143.1	10.3	95.0	18.4	73.0
Carbendazim	6.68	192.2	132.1	30.2	160.1	18.2	68.8
Methomyl	6.94	163.1	88.0	10.3	106.1	10.3	32.7
Thiamethoxam	7.47	292.0	181.1	20.7	211.1	12.9	81.4
Thiabendazole	7.73	202.0	175.0	19.2	131.1	27.8	93.8
Dicrotophos	8.45	238.1	112.0	10.3	127.0	11.4	56.2
Imidacloprid	8.85	255.9	175.1	19.0	209.1	19.6	77.0
Dimethoate	9.31	230.0	125.1	21.6	199.0	10.3	51.0
Acetamiprid	9.63	223.1	90.0	32.7	126.1	20.2	64.1
Thiacloprid	10.51	253.2	90.1	35.2	126.1	21.8	76.5
Simazine	12.06	202.1	124.0	17.7	132.1	21.1	82.7
Proposur	12.06	210.2	111.0	10.3	93.0	19.4	42.9
Imazalil	12.50	297.1	158.9	18.8	201.0	12.4	85.1
Atrazine	13.46	216.1	104.0	28.4	174.1	16.8	65.8
Fenpropimorph	13.53	304.3	147.1	23.0	117.1	47.8	90.8
Flutriafol	13.63	302.2	70.0	15.9	123.0	25.1	106.4
Metalaxyl	13.81	280.2	160.1	23.1	220.1	13.3	61.1
Diuron	13.97	233.1	72.0	18.7	160.0	27.6	75.0
Bupirimate	14.55	317.2	166.0	19.3	237.2	12.9	87.3
Azoxystrobin	14.73	404.2	344.1	23.9	372.1	13.9	60.9
Therbuthylazine	14.88	230.2	104.1	31.3	174.1	17.2	67.8
Boscalid	15.02	343.1	140.0	14.6	112.0	31.5	109.3
Cyproconazole 1	15.27	292.2	70.0	19.1	125.1	29.0	68.1
Myclobutanil	15.40	290.2	70.1	19.6	126.0	32.4	68.1
Bromuconazole 1	15.44	376.0	158.9	24.6	123.0	52.3	100.9
Mepanipyrim	15.54	224.2	77.0	34.6	106.1	22.8	95.5
Triazophos	15.54	314.1	162.1	14.0	119.0	27.4	79.2
Cyproconazole 2	15.61	292.2	70.0	19.1	125.1	29.0	68.1
Fluquinconazole	15.65	376.0	349.0	15.5	306.9	22.6	105.9
Fenhexamid	15.67	302.2	55.2	34.2	97.1	22.9	98.7
Epoxiconazole	15.92	330.2	121.0	16.3	101.0	35.1	86.9
Prochloraz	16.08	376.1	307.9	10.3	265.9	10.3	74.2
Rotenone	16.13	395.2	192.1	24.9	213.1	23.0	102.7
Bromuconazole 2	16.27	376.0	158.9	24.6	123.0	52.3	100.9
Pirimiphos methyl	16.38	306.2	108.0	30.9	164.1	22.2	91.3
Penconazole	16.41	284.2	70.1	17.5	159.0	28.7	72.0
Tebuconazole	16.50	308.2	70.1	23.5	125.0	36.9	83.1
Diazinon	16.57	305.2	153.1	21.1	169.1	20.6	83.6
Propiconazole	16.60	342.1	159.0	29.2	69.1	18.0	95.5
Chlorfenvinphos	16.61	359.1	155.1	12.0	169.9	39.8	80.2
Bitertanol	16.91	338.2	70.0	13.0	99.0	10.3	66.3
Pencycuron	17.07	329.2	125.0	20.2	89.0	47.9	86.4
Diniconazole	17.13	326.2	70.0	21.9	158.9	26.7	96.0
DIfenoconazole	17.15	406.2	251.1	24.3	336.9	16.0	73.8
Buprofezin	17.25	306.2	116.1	15.2	201.2	14.1	61.4
Trifloxystrobin	17.25	409.2	186.0	17.7	145.1	42.2	61.9
Profenofos	17.64	373.0	302.8	14.6	344.8	10.3	104.7
Tebufenpyrad	17.84	334.2	117.0	31.1	145.1	21.1	103.4
Pyriproxifen	18.08	322.2	96.0	15.8	185.2	23.7	66.3
Quinoxyfen	18.11	308.0	196.9	27.7	162.0	40.7	99.7
Fenazaquin	19.13	307.2	57.0	18.7	161.1	12.1	80.2

Table S2. Main mass spectral features of the pesticides analyzed in positive ion mode using ESI, APCI, and FµTP (helium, argon-propane, argon). The most abundant ion is shown in bold type

	**ESI**			**APCI/ FµTP (He, Ar-Prop, Ar)**	
**Compound**	**[M+H]+**	**[M+Na]+**	**Fragments**	**[M+H]+**	**Fragments**
Acephate	184.0	205.9	**143.0**, 161.1, 175.1	184.0	**143.0**, 161.1, 175.1
Carbendazim	**192.1**		160.0	**192.1**	160.0
Methomyl	163.1	**184.9**		**163.1**	
Thiamethoxam	292.0	**313.9**	211.0	292.0	211.1, **248.0**
Thiabendazole	**202.0**			**202.0**	
Dicrotophos	238.1	**260.0**	270.1	**238.1**	
Imidacloprid	256.0	**278.0**	175.1, 209.1	**256.0**	175.1, 209.1
Dimethoate	**230.0**	252.0	157.0, 171.0, 199.0	**230.0**	157.0, 171.0, 199.0
Acetamiprid	**223.1**	245.1		**223.1**	
Thiacloprid	**253.1**	275.0		**253.1**	
Simazine	**202.1**			**202.1**	
Proposur	210.1	**232.0**	168.0	210.1	**168.0**
Imazalil	**297.0**			**297.1**	239.0
Atrazine	**216.1**			**216.1**	
Fenpropimorph	**304.2**			**304.2**	
Flutriafol	**302.1**			302.1	**284.1**
Metalaxyl	280.1	**302.1**		**280.1**	
Diuron	**233.0**	254.9		**233.0**	
Bupirimate	**317.2**	339.1		**317.2**	
Azoxystrobin	404.2	426.1	344.2, **372.1**	404.2	344.2, **372.1**
Therbuthylazine	**230.1**		174.0	**230.1**	174.0
Boscalid	**343.0**	365.0		**343.0**	
Cyproconazole 1	**292.1**			**292.1**	
Myclobutanil	**289.1**			**289.1**	
Bromuconazole 1	**375.9**			**375.9**	
Mepanipyrim	**224.1**			**224.1**	
Triazophos	**314.1**	336.0		**314.1**	
Cyproconazole 2	**292.1**			**292.1**	
Fluquinconazole	**375.9**			**376.0**	
Fenhexamid	**302.0**	324.0		**302.2**	
Epoxiconazole	**330.1**			**330.1**	
Prochloraz	376.1	397.9	**307.9**, 339.9	376.0	**307.9**, 340.0
Rotenone	**395.1**	417.1		**395.1**	
Bromuconazole 2	**375.9**		296.1	**376.0**	296.1
Pirimiphos methyl	**306.1**			**306.1**	
Penconazole	**284.1**			**284.1**	
Tebuconazole	**308.1**			**308.2**	
Diazinon	**305.1**			**305.1**	
Propiconazole	**342.1**			**342.1**	
Chlorfenvinphos	358.9	**380.9**		**358.9**	
Bitertanol	338.9	**360.1**		**339.0**	
Pencycuron	**329.1**	351.1		**329.1**	
Diniconazole	**326.1**			**326.1**	
DIfenoconazole	**406.0**	428.0		**406.1**	
Buprofezin	**306.1**			**306.1**	
Trifloxystrobin	409.1	**431.1**		**409.1**	
Profenofos	**372.9**	394.8		**373.0**	
Tebufenpyrad	**334.1**	356.1		**334.1**	
Pyriproxifen	**322.1**	344.1		**322.1**	
Quinoxyfen	**307.9**			**308.0**	
Fenazaquin	**307.1**			**307.2**	

Table S3. Limits of quantification for ESI-amenable pesticides in the different food matrices analyzed in the study.

	LOQ apple (µg Kg^{-1})						LOQ grape (µg Kg^{-1})						LOQ avocado (µg Kg^{-1})					
**Compound**	**ESI**	**FµTP (He)**	**FµTP (Ar-Prop)**	**FµTP (Ar)**	**APCI**	**LMR (µg Kg^{-1})**	**ESI**	**FµTP (He)**	**FµTP (Ar-Prop)**	**FµTP (Ar)**	**APCI**	**LMR (µg Kg^{-1})**	**ESI**	**FµTP (He)**	**FµTP (Ar-Prop)**	**FµTP (Ar)**	**APCI**	**LMR (µg Kg^{-1})**
Acephate	25	25	25	25	250	10	25	25	25	25	250	10	83.3	83.3	83.3	83.3	833.3	10
Carbendazim	0.25	0.5	0.5	0.5	2.5	200	0.25	0.5	0.5	0.5	2.5	300	0.8	1.7	1.7	1.7	8.3	100
Methomyl	2.5	2.5	2.5	2.5	25	10	2.5	2.5	2.5	2.5	25	10	8.3	8.3	8.3	8.3	83.3	10
Thiamethoxam	2.5	0.5	2.5	2.5	5	300	2.5	0.5	2.5	2.5	5	400	8.3	1.7	8.3	8.3	16.7	500
Thiabendazole	0.25	0.5	0.5	0.5	2.5	4000	0.25	0.5	0.5	0.5	2.5	10	0.8	1.7	1.7	1.7	8.3	20000
Dicrotophos	0.5	2.5	2.5	2.5	5	-	0.5	2.5	2.5	2.5	5	-	1.7	8.3	8.3	8.3	8.3	-
Imidacloprid	2.5	0.25	0.25	0.25	2.5	100	2.5	0.25	0.25	0.25	2.5	700	8.3	0.8	0.8	1.7	8.3	10
Dimethoate	0.25	0.25	0.25	0.25	2.5	10	0.5	0.25	0.25	0.25	2.5	10	1.7	1.7	0.8	0.8	8.3	10
Acetamiprid	0.25	0.25	0.25	0.25	5	400	0.25	0.25	0.25	0.25	2.5	500	1.7	0.8	0.8	1.7	8.3	10
Thiacloprid	0.25	0.25	0.25	0.25	2.5	300	0.25	0.25	0.25	0.25	2.5	10	1.7	0.8	0.8	0.8	8.3	10
Simazine	2.5	2.5	2.5	2.5	5	10	2.5	2.5	2.5	2.5	5	200	8.3	8.3	8.3	0.8	16.7	10
Proposur	2.5	0.5	2.5	2.5	25	5	2.5	0.5	2.5	2.5	25	5	8.3	1.7	8.3	8.3	83.3	10
Imazalil	0.5	2.5	2.5	2.5	5	10	0.5	2.5	2.5	2.5	25	10	1.7	8.3	8.3	8.3	83.3	10
Atrazine	0.25	0.25	0.5	0.25	2.5	50	0.25	0.25	0.25	0.25	2.5	50	0.8	0.8	0.8	0.8	8.3	50
Fenpropimorph	0.25	0.25	0.25	0.25	2.5	10	0.25	0.25	0.25	0.25	2.5	10	0.8	1.7	0.8	1.7	8.3	10
Flutriafol	0.5	0.5	0.5	0.5	5	400	0.5	0.5	0.5	0.5	2.5	800	1.7	1.7	1.7	1.7	8.3	10
Metalaxyl	0.25	0.25	0.25	0.25	2.5	1000	0.25	0.25	0.25	0.25	2.5	2000	0.8	0.8	0.8	0.8	8.3	10
Diuron	2.5	2.5	2.5	2.5	25	10	2.5	2.5	2.5	2.5	25	10	8.3	8.3	8.3	8.3	83.3	10
Bupirimate	0.5	2.5	2.5	2.5	5	300	0.5	2.5	2.5	2.5	5	1500	1.7	8.3	8.3	8.3	16.7	10
Azoxystrobin	0.25	0.25	0.25	0.25	2.5	10	0.25	0.25	0.25	0.25	2.5	3000	0.8	0.8	0.8	0.8	8.3	10
Therbuthylazine	0.25	0.25	0.5	0.25	2.5	10	0.25	0.25	0.5	0.25	2.5	10	0.8	0.8	1.7	0.8	8.3	10
Boscalid	2.5	2.5	2.5	2.5	25	2000	5	2.5	2.5	2.5	25	5000	8.3	8.3	8.3	8.3	83.3	10
Cyproconazole 1	2.5	2.5	2.5	2.5	5	100	2.5	2.5	2.5	2.5	5	200	8.3	8.3	8.3	8.3	16.7	50
Myclobutanil	2.5	2.5	2.5	2.5	25	600	2.5	2.5	2.5	2.5	25	1500	8.3	8.3	8.3	8.3	83.3	10
Bromuconazole 1	2.5	2.5	2.5	2.5	25	10	2.5	2.5	2.5	2.5	25	10	8.3	8.3	8.3	8.3	16.7	10
Mepanipyrim	0.25	0.5	0.5	0.5	2.5	10	0.25	0.5	0.5	0.5	2.5	2000	0.8	1.7	1.7	1.7	8.3	10
Triazophos	0.25	0.5	0.5	0.5	2.5	10	0.25	0.5	0.5	0.5	2.5	10	0.8	1.7	1.7	1.7	8.3	10
Cyproconazole 2	2.5	2.5	2.5	2.5	5	100	2.5	2.5	2.5	2.5	5	200	8.3	8.3	8.3	8.3	16.7	50
Fluquinconazole	2.5	0.5	0.5	0.5	5	10	2.5	0.5	0.5	0.5	25	10	8.3	1.7	1.7	1.7	83.3	10
Fenhexamid	0.5	0.5	0.5	0.5	5	10	2.5	0.5	0.5	0.5	5	15000	1.7	1.7	1.7	1.7	16.7	10
Epoxiconazole	0.25	0.25	0.25	0.25	2.5	10	0.25	0.25	0.25	0.25	2.5	10	0.8	0.8	0.8	0.8	8.3	10

Table S3. Limits of quantification for ESI-amenable pesticides in the different food matrices analyzed in the study (continuation)

	LOQ apple (µg Kg^{-1})						LOQ grape (µg Kg^{-1})						LOQ avocado (µg Kg^{-1})					
**Compound**	**ESI**	**FµTP (He)**	**FµTP (Ar-Prop)**	**FµTP (Ar)**	**APCI**	**LMR (µg Kg^{-1})**	**ESI**	**FµTP (He)**	**FµTP (Ar-Prop)**	**FµTP (Ar)**	**APCI**	**LMR (µg Kg^{-1})**	**ESI**	**FµTP (He)**	**FµTP (Ar-Prop)**	**FµTP (Ar)**	**APCI**	**LMR (µg Kg^{-1})**
Prochloraz	2.5	2.5	2.5	2.5	25	30	2.5	2.5	2.5	2.5	25	30	8.3	8.3	8.3	8.3	83.3	30
Rotenone	2.5	0.25	0.5	0.5	5	10	2.5	0.25	0.5	0.5	5	10	8.3	0.8	1.7	1.7	16.7	10
Bromuconazole 2	2.5	2.5	2.5	2.5	25	10	2.5	2.5	2.5	2.5	25	10	8.3	8.3	8.3	8.3	83.3	10
Pirimiphos methyl	0.25	0.5	0.5	0.5	2.5	10	0.25	0.5	0.5	0.5	2.5	10	0.8	1.7	1.7	1.7	8.3	10
Penconazole	0.25	0.25	0.25	0.25	2.5	150	0.25	0.25	0.25	0.25	2.5	500	0.8	0.8	0.8	0.8	8.3	10
Tebuconazole	0.5	0.5	0.5	0.5	5	300	2.5	0.5	0.5	0.5	5	500	8.3	1.7	1.7	1.7	16.7	20
Diazinon	0.25	0.25	0.25	0.25	2.5	10	0.25	0.25	0.25	0.25	2.5	10	0.8	0.8	0.8	0.8	8.3	10
Propiconazole	0.5	0.5	0.5	2.5	5	10	0.5	0.5	0.5	2.5	5	10	1.7	1.7	1.7	8.3	16.7	10
Chlorfenvinphos	0.5	0.5	0.5	2.5	5	10	0.5	0.5	2.5	2.5	5	10	1.7	1.7	1.7	8.3	16.7	10
Bitertanol	5	2.5	2.5	5	25	10	5	2.5	2.5	5	25	10	16.7	8.3	8.3	16.7	83.3	10
Pencycuron	0.25	0.25	0.25	0.25	2.5	20	0.25	0.25	0.25	0.25	2.5	20	0.8	0.8	0.8	0.8	8.3	20
Diniconazole	0.5	0.5	2.5	2.5	25	10	0.5	0.5	2.5	2.5	5	10	1.7	1.7	8.3	8.3	16.7	10
DIfenoconazole	2.5	2.5	2.5	2.5	25	800	2.5	2.5	2.5	2.5	25	3000	8.3	8.3	8.3	8.3	83.3	600
Buprofezin	0.25	0.5	0.5	0.5	2.5	10	0.25	0.5	0.5	0.5	2.5	10	0.8	1.7	1.7	1.7	8.3	10
Trifloxystrobin	0.25	0.25	0.25	0.25	2.5	700	0.25	0.25	0.25	0.25	2.5	3000	0.8	0.8	0.8	0.8	8.3	10
Profenofos	2.5	0.5	2.5	2.5	25	10	2.5	0.5	2.5	2.5	25	10	8.3	1.7	8.3	8.3	83.3	10
Tebufenpyrad	0.25	0.25	0.25	0.25	2.5	300	0.25	0.25	0.25	0.25	2.5	600	0.8	0.8	0.8	0.8	8.3	10
Pyriproxifen	0.25	0.5	0.5	0.5	5	50	0.25	0.5	0.5	0.5	5	10	0.8	1.7	1.7	1.7	16.7	10
Quinoxyfen	0.25	0.5	0.5	0.5	2.5	50	0.25	0.5	0.5	0.5	2.5	1000	0.8	1.7	1.7	1.7	8.3	20
Fenazaquin	0.5	0.5	0.5	0.5	2.5	150	0.5	0.5	0.5	0.5	2.5	10	1.7	1.7	1.7	8.3	8.3	10

Table S4. Intra-day and inter-day precision (% RSD) for ESI-amenable pesticides using the FµTP ionization source at a concentration level of 1 µg L^{-1}

	Solvent		Apple		Grape		Avocado	
**Compound**	**Intra-day (% RSD) (n=6)**	**Inter-day (% RSD) (n=5)**	**Intra-day (% RSD) (n=6)**	**Inter-day (% RSD) (n=5)**	**Intra-day (% RSD) (n=6)**	**Inter-day (% RSD) (n=5)**	**Intra-day (% RSD) (n=6)**	**Inter-day (% RSD) (n=5)**
Acephate	6.2	3.6	3.7	6.4	5.0	10.1	2.4	5.8
Carbendazim	1.8	9.6	2.6	4.7	2.8	7.6	2.6	5.3
Methomyl	2.8	7.8	3.0	4.1	3.3	9.9	3.4	9.7
Thiamethoxam	1.2	5.0	3.5	3.6	4.0	4.6	2.2	8.2
Thiabendazole	1.4	10.7	2.6	8.5	3.2	9.7	2.1	8.9
Dicrotophos	3.1	6.7	4.3	9.9	4.0	10.6	2.0	9.7
Imidacloprid	2.7	6.2	4.5	3.1	1.7	5.2	1.9	9.2
Dimethoate	1.2	9.3	3.2	6.4	8.4	11.9	2.8	7.7
Acetamiprid	1.7	7.9	3.9	6.3	2.4	4.9	1.9	7.0
Thiacloprid	0.6	8.0	2.4	3.5	2.8	5.2	2.9	9.5
Simazine	3.0	6.6	3.3	5.6	2.8	3.9	1.9	5.7
Proposur	0.5	11.5	3.2	6.0	2.2	6.8	2.1	9.0
Imazalil	6.2	7.9	9.3	9.5	7.0	9.7	5.4	4.7
Atrazine	1.3	8.7	3.3	5.5	4.0	5.2	3.8	7.2
Fenpropimorph	7.6	7.5	9.2	8.1	6.5	9.1	3.5	10.1
Flutriafol	2.7	7.7	2.7	1.2	2.9	7.1	3.0	10.3
Metalaxyl	1.8	7.4	1.4	2.7	3.0	5.8	2.8	11.9
Diuron	2.2	9.6	3.4	3.6	3.4	4.2	2.4	8.2
Bupirimate	4.4	3.1	6.1	5.6	6.5	7.6	4.6	8.5
Azoxystrobin	4.3	5.4	2.7	6.0	4.4	3.4	3.1	12.7
Therbuthylazine	4.7	7.7	4.7	4.1	3.2	7.1	3.8	8.6
Boscalid	3.9	12.2	3.5	3.8	3.7	4.3	3.3	6.7
Cyproconazole 1	2.0	9.1	3.9	5.3	5.0	5.1	1.6	9.7
Myclobutanil	2.4	6.0	2.8	3.1	5.1	5.5	1.9	6.0
Bromuconazole 1	2.3	6.4	3.2	2.6	4.0	7.5	6.7	9.8
Mepanipyrim	7.3	6.6	9.4	10.6	4.6	11.3	5.9	5.9
Triazophos	5.9	7.2	2.9	6.0	4.6	7.2	4.8	8.5
Cyproconazole 2	2.6	8.0	4.6	3.5	2.8	4.2	2.1	6.6
Fluquinconazole	3.8	13.8	4.4	3.0	2.4	1.1	4.8	12.2
Fenhexamid	3.0	14.1	4.1	4.5	3.6	8.8	6.7	5.8
Epoxiconazole	2.4	7.6	4.9	5.2	2.1	7.6	2.0	7.9
Prochloraz	3.5	6.9	5.7	4.6	4.4	10.4	5.5	11.7

Table S4. Intra-day and inter-day precision (% RSD) for ESI-amenable pesticides using the FµTP ionization source at a concentration level of 1 µg L^{-1} (continuation)

	Solvent		Apple		Grape		Avocado	
**Compound**	**Intra-day (% RSD) (n=6)**	**Inter-day (% RSD) (n=5)**	**Intra-day (% RSD) (n=6)**	**Inter-day (% RSD) (n=5)**	**Intra-day (% RSD) (n=6)**	**Inter-day (% RSD) (n=5)**	**Intra-day (% RSD) (n=6)**	**Inter-day (% RSD) (n=5)**
Rotenone	3.4	4.9	8.9	7.1	3.4	7.1	4.0	9.7
Bromuconazole 2	4.5	3.6	5.2	3.8	2.5	4.7	3.8	8.9
Pirimiphos methyl	5.6	10.8	4.5	8.1	8.6	11.1	3.8	9.5
Penconazole	3.2	4.3	2.6	3.4	2.6	2.6	3.4	11.6
Tebuconazole	2.6	7.1	5.4	6.5	3.1	6.8	5.3	8.7
Diazinon	3.3	10.9	5.1	7.8	5.7	7.4	5.5	8.1
Propiconazole	2.7	6.0	2.6	3.4	2.9	1.9	1.7	9.4
Chlorfenvinphos	3.6	9.4	4.6	5.2	2.4	5.0	4.3	9.4
Bitertanol	2.1	6.3	9.6	7.1	1.7	7.3	1.9	8.8
Pencycuron	4.7	5.8	4.3	1.1	3.6	6.6	5.6	9.9
Diniconazole	4.1	6.4	3.8	5.6	3.5	5.4	4.5	12.6
DIfenoconazole	2.1	7.0	6.3	6.0	2.3	9.2	2.4	7.7
Buprofezin	9.9	7.3	6.4	7.7	3.5	11.5	6.4	11.5
Trifloxystrobin	3.6	5.2	3.6	12.7	3.0	7.8	3.6	9.7
Profenofos	3.8	6.2	6.8	7.4	2.8	3.4	5.3	4.4
Tebufenpyrad	2.0	8.3	7.2	5.5	2.0	1.6	2.5	6.4
Pyriproxifen	4.0	13.2	8.6	8.6	3.6	7.7	5.8	8.2
Quinoxyfen	2.8	2.8	10.1	6.9	1.9	3.4	4.3	7.6
Fenazaquin	2.2	9.1	7.7	14.0	1.0	5.5	3.3	9.7

Table S5. Intra-day and inter-day precision (% RSD) for organochlorine pesticides using the FμTP ionization source at a concentration level of 50 μg L^{-1}

	Solvent		Apple		Grape		Avocado	
**Compound**	**Intra-day (% RSD) (n=6)**	**Inter-day (% RSD) (n=5)**	**Intra-day (% RSD) (n=6)**	**Inter-day (% RSD) (n=5)**	**Intra-day (% RSD) (n=6)**	**Inter-day (% RSD) (n=5)**	**Intra-day (% RSD) (n=6)**	**Inter-day (% RSD) (n=5)**
Chlorothalonil	6.3	8.7	5.5	6.3	6.2	13.4	8.4	7.3
Dicofol	8.8	7.7	8.0	4.2	9.4	9.5	5.6	13.9
α-Endosulfan	7.7	12.2	9.3	6.3	11.5	16.5	8.1	18.3
β-Endosulfan	6.9	11.4	9.0	12.2	7.9	18.4	6.9	13.7
Endosulfan sulfate	4.5	11.7	4.2	9.1	9.0	12.6	7.5	15.3
Pentachlorobenzene	8.8	14.6	4.5	9.9	5.5	10.2	6.9	10.1
Hexachlorobenzene	7.7	8.0	5.6	10.2	9.9	14.6	6.4	6.8
Captafol	5.1	14.9	5.6	6.9	8.3	10.6	7.3	11.5
Folpet	2.5	13.7	5.5	8.0	5.4	5.2	6.2	6.4
Captan	7.9	12.7	5.0	12.6	7.3	12.0	7.7	12.8
Quintozene	2.4	3.9	5.7	5.0	6.4	9.0	7.9	10.3
Chlorpyrifos ethyl	6.7	13.0	9.3	8.2	11.3	12.8	8.9	31.6
